# Microbiome characterization of alpine water springs for human consumption reveals site- and usage-specific microbial signatures

**DOI:** 10.3389/fmicb.2022.946460

**Published:** 2022-10-05

**Authors:** Renato Pedron, Alfonso Esposito, William Cozza, Massimo Paolazzi, Mario Cristofolini, Nicola Segata, Olivier Jousson

**Affiliations:** ^1^Department of Cellular, Computational and Integrative Biology – CIBIO, University of Trento, Trento, Italy; ^2^International Centre for Genetic Engineering and Biotechnology – ICGEB, Trieste, Italy; ^3^Agenzia provinciale per la protezione dell'ambiente – APPA, Trento, Italy; ^4^Istituto G.B. Mattei, Stenico, Italy

**Keywords:** microbiome, water springs, 16S amplicon sequencing, longitudinal study, human consumption and production

## Abstract

The microbiome of water springs is gaining increasing interest, especially in water intended for human consumption. However, the knowledge about large-scale patterns in water springs microbiome is still incomplete. The presence of bacteria in water sources used for human consumption is a major concern for health authorities; nonetheless, the standard microbiological quality checks are focused only on pathogenic species and total microbial load. Using 16S rRNA high throughput sequencing, we characterized the microbiome from 38 water springs in Trentino (Northern Italy) for 2 consecutive years in order to gain precious insights on the microbiome composition of these unexplored yet hardly exploited environments. The microbiological studies were integrated with standard measurements of physico-chemical parameters performed by the Provincial Office for Environmental Monitoring in order to highlight some of the dynamics influencing the microbial communities of these waters. We found that alpha diversity showed consistent patterns of variation overtime, and showed a strong positive correlation with the water nitrate concentration and negatively with fixed residue, electrical conductivity, and calcium concentration. Surprisingly, alpha diversity did not show any significant correlation with neither pH nor temperature. We found that despite their remarkable stability, different water springs display different coefficients of variation in alpha diversity, and that springs used for similar purposes showed similar microbiomes. Furthermore, the springs could be grouped according to the number of shared species into three major groups: low, mid, and high number of shared taxa, and those three groups of springs were consistent with the spring usage. Species belonging to the phyla Planctomycetes and Verrucomicrobia were prevalent and at relatively high abundance in springs classified as low number of shared species, whereas the phylum Lentisphaerae and the Candidate Phyla radiation were prevalent at higher abundance in the mineral and potable springs. The present study constitutes an example for standard water spring monitoring integrated with microbial community composition on a regional scale, and provides information which could be useful in the design and application of future water management policies in Trentino.

## Introduction

The microbiome of water springs has been deeply explored by a large number of studies, however, the majority of them focused on extreme environments (Power et al., 2018; Roy et al., 2020). Often, such studies had the intent of identifying novel bacterial species or understanding the impact of environmental parameters on microbial populations. Springs that provide water destined for human consumption have been instead scarcely investigated (Hull et al., 2019; Varga, 2019; Miller et al., 2021). The microbiome of drinking water has been explored mainly to describe spatial and temporal taxonomic compositional patterns, or to investigate the effects of distribution and purification plants on water quality and safety (Bruno et al., 2018), leaving open questions on the native microorganisms (Savio et al., 2018). The presence of bacteria in water sources used for human consumption is a major concern for health authorities and the management of microorganisms for water safety is strictly regulated, but it is focused only on total microbial load and on seeking for the presence of pathogenic species that represent a minor fraction of the microbiome diversity. Along with bacteria, chemical contamination is another issue of increasing concern, the main contaminants deriving from human activities include chlorinated solvents, petroleum hydrocarbons, heavy metals, pesticides, human and veterinary pharmaceuticals, and personal care products (Burri et al., 2019). Surface waters are the most exposed compartment to this risk, whereas groundwaters are more protected by a physical shield constituted by layers of active soil and sediments, and groundwater microbes have the potential to attenuate the contaminants and prevent their accumulation in the environment (Kolvenbach et al., 2014).

There are many geographical areas where treatment and disinfection efforts of raw water are intentionally kept at a minimum to meet the consumer’s desire for high-quality, safely consumable drinking water without a noticeable change in taste as a result of high chlorine concentrations for disinfection. Usually, such areas are in proximity with districts where groundwater is better preserved and the water quality itself is higher [e.g., the Alps (Griebler and Avramov, 2015), or the Netherlands (Roeselers et al., 2015)]. In areas where drinking water abstraction relies on surface waters, instead, water must be treated, one of the most common methods is bank filtration; however, this procedure results in carryover of pathogens and chemicals, thus making necessary further treatments to improve water quality (Griebler and Avramov, 2015). Such treatments constitute a disturbance for the native water microbiome (Zhang et al., 2017), furthermore, it has been shown that even disinfection with either activated carbon filters or chlorination, did not succeed in removing the abundant fraction known as “microbial dark matter” (Bruno et al., 2017). Therefore, it is crucial to gather a deep knowledge on the microbiome composition of water springs used for human consumption.

The study of potable water microbiome is also relevant for the European Council Directive 80/777/EEC on the approximation of the laws of the Member States relating to the exploitation and marketing of natural mineral waters. The directive states that: “Any disinfection treatment … and … the addition of bacteriostatic elements or any other treatment likely to change the viable colony count of the natural mineral water shall be prohibited.” Thus the spring microbiome remains unaltered and is consequently present in bottled water. The same principle of leaving intact the microbiome of water applies also to Spa and Medical Spa. Spring water used for medical treatments has been investigated in order to unravel the curative properties of balneotherapy (Pedron et al., 2019; Aburto-Medina et al., 2020; Bourrain et al., 2020) expressing a growing interest in microbial communities of spring waters for human use.

The stability of the environmental conditions in groundwaters results in a temporally stable community of macroorganisms (stygobiont). This stability is a well-acknowledged fact, and previous studies suggest that it also applies to microbial communities (Farnleitner et al., 2005). However, there is evidence suggesting a role of seasonality in shaping groundwater microbiome, as a karst aquifer study showed that the bacterial community is taxonomically stable over time but discharge-responsive (Savio et al., 2019). These circumstances translate into a difficulty in understanding the ecological role and the dynamics of these communities and, subsequently, the metabolic processes that they carry out. Due to their oligotrophic nature and to the absence of direct light, these ecological niches have shown a high potential for peculiar metabolic processes; these include for instance chemolithoautotrophic processes like denitrification linked to the oxidation of reduced sulfur compounds, as well as the capacity for anaerobic ammonium oxidation in limestone aquifers (Opitz et al., 2014; Herrmann et al., 2015; Kumar et al., 2017). Such processes are coupled with the fixation of inorganic carbon to build up the groundwater microbial biomass (Nowak et al., 2017; Schwab et al., 2017).

The province of Trento (Trentino), in northern Italy, is an alpine water-rich region and it hosts 4 water bottling companies and seven medical spas. The principles of minimal treatment of potable water are applied to the potable water supply network. In this work, we present a seasonal characterization for 2 consecutive years of the microbiomes of 38 Trentino water springs that are destined for human consumption for one of the following purposes: municipal tap water, Spa, and bottled water. We carried our sampling in parallel with the standard water quality check performed by the Provincial Office for Environmental Monitoring in order to intersect our findings with a panel of environmental metadata. The aim of this work is to describe the diversity patterns in space and time in relation to water usage, more specifically, we intend to answer the following questions: (i) how does the water spring microbiome diversity vary across the spatial and temporal scale? (ii) Is there any chemical or physical parameter that correlates with any feature of the microbiome? (iii) How do the Trentino water springs resemble each other?

The answers to those questions are useful in drawing guidelines to define the policies for conservation and usage of the water and for biomonitoring purposes.

## Materials and methods

### Experimental design, water sampling, and environmental parameters measurements

**Figure 1:**
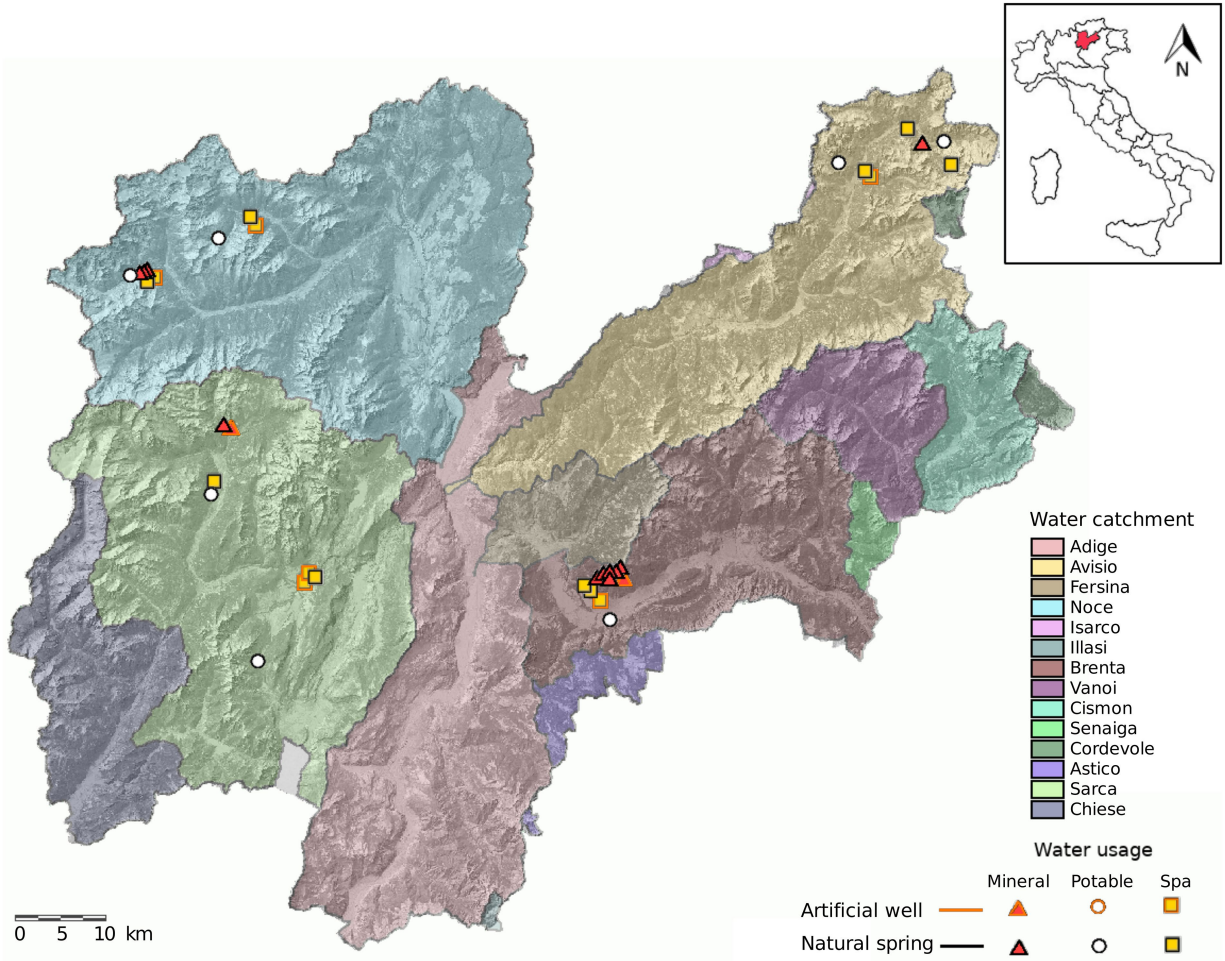
Map of Trentino and location of the sampled springs. The sampling design consisted of sampling 38 water springs distributed over four water catchments in Trentino. We included springs used for bottling (mineral), municipal tap water (potable) and SPA.

The springs were sampled one time in each season for two consecutive years (2018 and 2019) by the Trentino Health Agency (Azienda Provinciale per i Servizi Sanitari di Trento, APSS) during routine spring water safety controls (Figure 1; Supplementary Table 1). Samples were collected as single replicates in 4 l sterile bottles and kept at air temperature (about 20°C) during transportation to the laboratory. All samples were collected from monitor taps at the water intake structure at the spring, the tap was sterilized by flame and the water was let flow for 1 min before sampling.

Every sample was processed within 8 h since collection. The water was then filtered using 0.22 μm Pall Supor filters (Pall Corporation, Port Washington, NY, United States). At the same time a sample of 1 liter was collected for the chemical analysis performed by Trentino Environmental Agency (Agenzia Provinciale per la Protezione dell’Ambiente, APPA). Measured chemical parameters were: total hardness as French degrees (°f), pH, electrical conductivity as microsiemens per centimeter (μS/cm), oxidizability (O2), fixed residue, antimony (Sb), arsenic (As), selenium (Se), fluoride (F-), chloride (Cl-), bromide (Br-), nitrate (NO3-), sulfate (SO42-), sodium (Na), potassium (K), lithium (Li), calcium (Ca), magnesium (Mg), strontium (Sr), aluminum (Al), barium (Ba), boron (B), cadmium (Cd), chrome (Cr), iron (Fe), phosphor (P), manganese (Mn), nichel (Ni), lead (Pb), copper (Cu), vanadium (V), zinc (Zn), mercury (Hg), sulfur (S-), silicon dioxide (SiO2), ammonium (NH4+), nitrite (NO2-), bicarbonate (HCO3-), and free carbon dioxide (CO2) as milligrams per liter (mg/l). Measured chemical parameters, testing method, and certified testing procedure are available in Supplementary Table 2.

### DNA extraction and 16S rRNA sequencing

Total DNA was extracted using RapidWater DNA extraction kit (MoBio, Carlsbad, CA, United States) with minor modifications: at step 5 of the protocol, the PowerWater Beat Tube was heated at 65°C for 10 min and mechanical cell lysis was extended to 10 min for all samples. All other steps were performed following the manufacturer’s instructions. The V4 hypervariable region of the 16S rRNA gene was amplified by PCR, using the primer pairs 515F/806R (Caporaso et al., 2012), with the 5PRIME HotMasterMix (Quanta BIO, Beverly, MA, United States). The length of the amplicons was 253 bp. Negative controls were included during sampling and main wet-lab steps, including PCR blanks. Amplicons concentration, size range, and purity were measured using Agilent high sensitivity (HS) DNA kit on the Bioanalyzer 2,100 (Agilent Technologies Italia S.p.A, Milano, Italy). Based on the molarity estimated using Bioanalyzer, each PCR product was diluted before pooling. The final pool was purified using the Agen-court AMPure XP DNA purification kit (Beckman Coulter, Brea, CA, USA), following the manufacturer’s instructions. Amplicons were sequenced on an Illumina MiSeq platform with 2 × 300 bp paired-end protocol. The data are openly available in NCBI Sequence Read Archive with the accession number PRJNA837552.

### Bioinformatics analyses

The fastq files were quality checked using FastQC (Andrews, 2010), and initial sequence analysis was performed with QIIME2 (Bolyen et al., 2019); demultiplexing, denoising, read pairs merging, and feature table construction were performed using DADA2 (Callahan et al., 2016). Taxonomy assignment was performed using sklearn and sepp fragment insertion using the SILVA database (Mirarab et al., 2012; Quast et al., 2013).

Diversity analyses were performed using QIIME2 on a rarefied dataset (30,000 reads) to prevent statistical artifacts due to different sequencing depths. The alpha diversity was measured with the Shannon index, as it is well suited to the nature of our dataset, which consists of multiple samples with low biomass and a high number of unknown species. Significant differences of Shannon index among the springs in space and time were determined using the Wilcoxon test, to better determine which springs showed the highest variation in terms of alpha diversity we calculated the coefficient of variation of Shannon’s index over time. To perform beta diversity analyses, we measured unweighted UniFrac metrics on the rarefied dataset, and displayed it through 2D PCoA (Lozupone et al., 2011). We selected this metric as it has recently been shown that the presence, rather than the abundance, maximizes the classification accuracy among the samples (Giliberti et al., 2022). We computed the pairwise distances between different samples from a single spring, and compared them with the pairwise distances between samples of Winter 2018, selected as control because it had the lowest average distance among all seasons.

The alpha diversity index was correlated with the content of Nitrates using the spearman correlation, and to determine correlations between the diversity index and all physico-chemical measures, we performed partial correlations, which accounted for autocorrelation among the variables.

All statistical correlations were calculated and created using R (R Foundation for Statistical Computing 2018), and ordination of the springs based on chemical parameters was performed using the Vegan package (Oksanen et al., 2019). All the plots were drawn using the ggplot2 R-package.

To calculate the degree of similarity among each pair of springs we calculated the number of shared Amplicon Sequence Variants (ASVs), the number of shared ASVs was plotted with a heatmap using the pheatmap R- package.

## Results

### Illumina sequencing results

The 267 samples were divided in 4 different sequencing runs. The sequencing produced a total of 53,978,996 paired-end reads. After the DADA2 step, there were an average of 165,950 ± 72,697.12 reads per sample with a median of 153,295 and a maximum number of 432,685 reads (Supplementary Table 3). Two samples with less than 30,000 reads were discarded from further analysis. After quality check, there were an average of 99,294.25 ± 36,385.04 reads (Supplementary Table 1).

### The diversity of spring microbiomes is affected by the seasonal cycle

**Figure 2:**
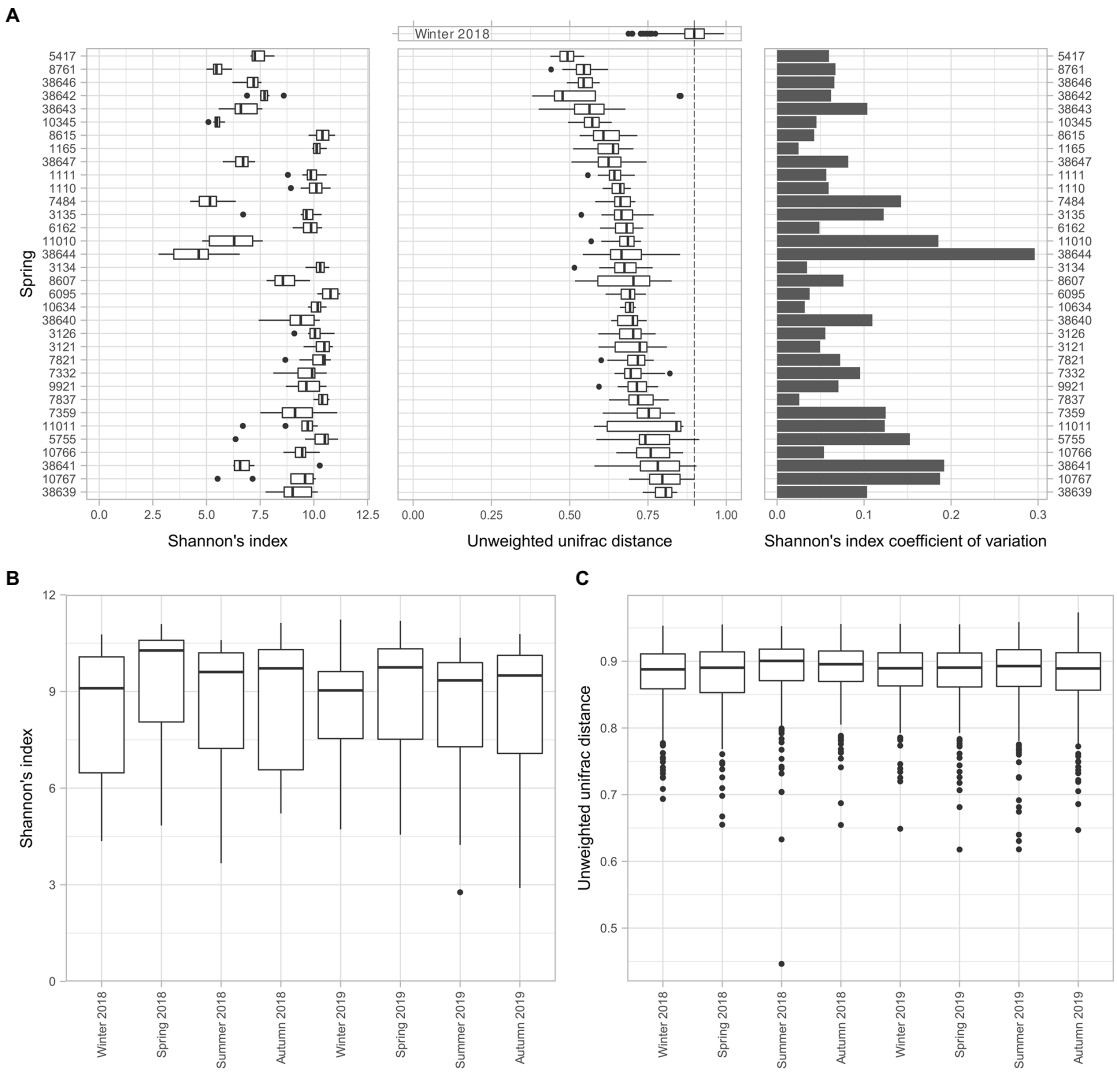
Evaluation of the microbiome stability of the springs. The perturbability of the microbiome has been evaluated for multiple aspects. **(A)** Boxplot of Shannon’s index alpha diversity values of all the springs, coupled with the boxplot of the pairwise unweighted uniFrac distance values of each spring among timepoints and the coefficient of variation of the Shannon’s alpha diversity index for each spring among sampling timepoints. **(B)** Boxplot of the Shannon’s alpha diversity index for each timepoint. **(C)** Boxplot of the pairwise unweighted uniFrac distance values of all the springs for each of the sampling timepoints.

The alpha and beta diversity analyses were performed to assess the perturbability of the microbiome. The alpha diversity analysis using Shannon’s index of each spring among the different time points revealed the heterogeneity of the dataset (Figure 2A). While some of the springs maintained an almost constant alpha diversity over the sampled time series, including springs 1,165, 3,134, and 7,837, other ones, for example, springs 38,644, 11,010, and 38,641, presented a larger distribution of data, probably connected to random factors. We found that the largest variation in Shannon’s index was in the springs 38,644 and 38,641, whereas the springs with the lowest coefficient of variations were 7,837 and 1,165 (Figure 2A).

The compositional shifts of the microbiome over time were evaluated through a beta diversity analysis. The unweighted uniFrac distances among all the time points for a given spring provide an alternative view of the stability of the microbiome composition over time. This analysis showed that the similarity of different samples of a single spring over time is significantly higher compared to a set of different springs, represented by measurements of winter 2018, used as control because it showed the lowest diversity among all seasons. At the same time, the data from the sampling taken in winter 2018 highlighted the differences in terms of consistency of the microbiome composition over time for different springs, without revealing appreciable patterns.

We could not identify clear patterns in the variations of taxonomic composition for any single spring over time. However, examining the whole set of springs over different time points highlighted differences in terms of alpha diversity (Figure 2B). The results evidenced that the alpha diversity during winter seasons is lower than the average while for spring seasons it resulted to be higher. A Wilcoxon test for paired samples between seasons (Supplementary Table 4) revealed significant differences between the spring seasons and the other ones, with exception of autumn.

Another way to assess the perturbability of the spring microbiomes is to evaluate changes in terms of beta diversity distances (Figure 2C). Using the univocal unweighted uniFrac distance between all springs in a single time point it is possible to evaluate a drift in microbial composition between seasons. In this regard, we did not detect any significant difference in the beta diversity among the springs in the different time points.

### Environmental parameters are consistent with microbial community composition

The chemical data was analyzed with a reciprocal correlation analysis (Supplementary Figure 1). The Spearman correlation scores allowed the clustering of chemical measures according to similar behavior to simplify downstream graphical representations.

Chemical measures such as electrical conductivity, fixed residue and concentration of major solutes like sodium (Na), chloride (Cl-), clustered together and maintained similar behavior with other measured parameters. The same was observed for other closely related elements like calcium and magnesium (that form dolomite as CaMg(CO3)2) and traces of heavy metals like chrome, mercury, and cadmium.

**Figure 3:**
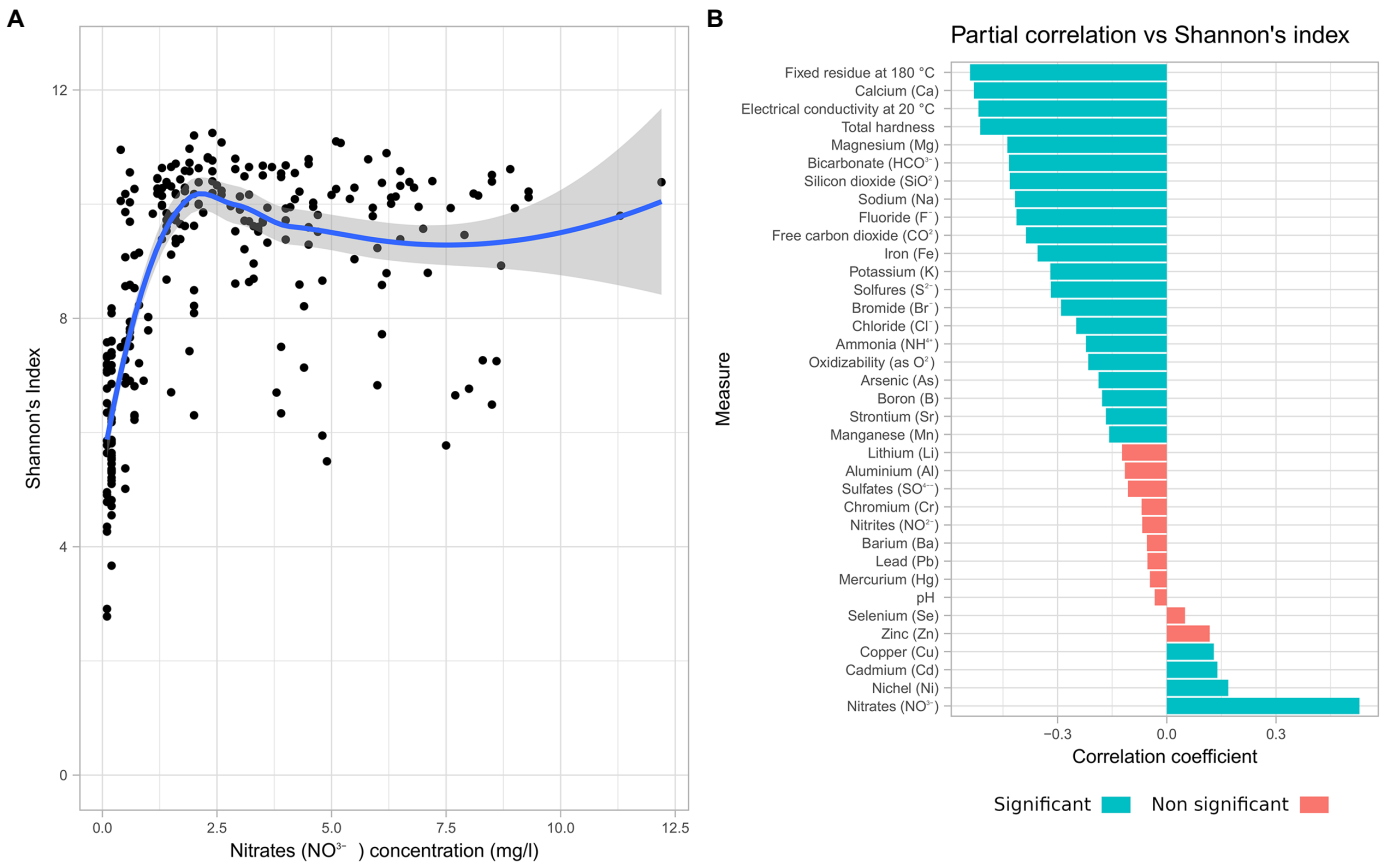
Nitrate concentration influences the microbiome diversity. Correlation of chemical data with alpha diversity. **(A)** Scatterplot of Shannon alpha diversity index of all samples in the study vs. Nitrate concentration. **(B)** Spearman partial correlations, given Nitrates, of all measured chemical parameters.

The chemical moieties in water and its physical parameters are *de facto* defining the environment hosting the microbiome. The Spearman correlation analysis between each of the measured physico-chemical parameters and Shannon’s index revealed that Nitrate concentration had a positive correlation (Spearman’s rho = 0.53) with alpha diversity (Figure 3A). For this reason, a partial correlation accounting for the effects on nitrate concentration was computed (Figure 3B).

Most of the chemical parameters measured were negatively correlated with increasing alpha diversity, with the exception of copper, cadmium, and nickel. While 24 of the measured parameters had a significant correlation with the alpha diversity, only 4 presented a coefficient value above 0.5. Among these are fixed residue, electrical conductivity, and total hardness, which are representative of the total quantity of solutes. Temperature and pH instead have shown a low impact on alpha diversity.

**Figure 4:**
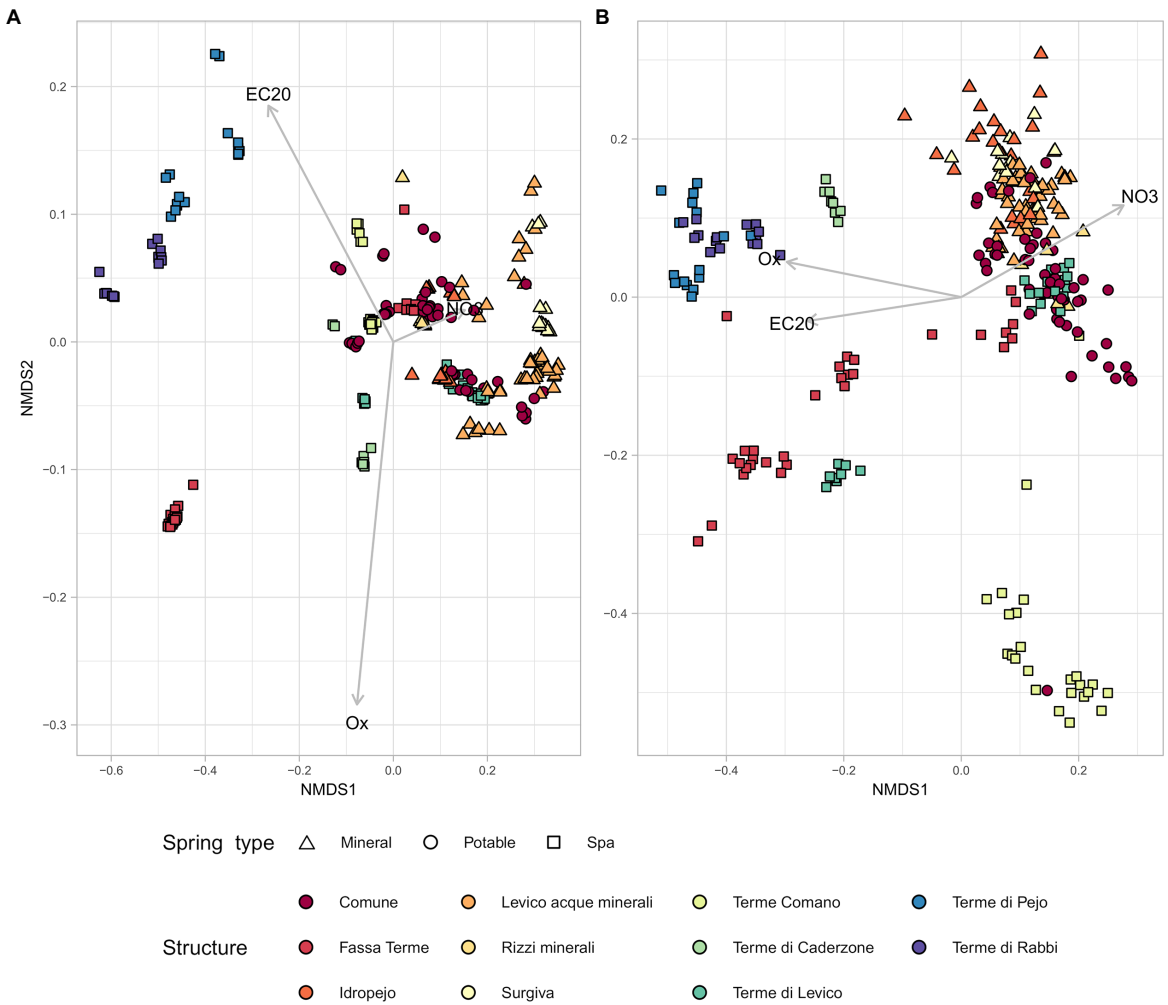
The physico-chemical parameters and the sequencing data present similar behavior in NMDS. NMDS plots based on unweighted Bray-Curtis metrics. Each symbol represents a sample, all samples are categorized by spring type and facility. **(A)** NMDS based on physico-chemical parameters. **(B)** NMDS based on sequencing data.

The interaction between the chemical parameters and the microbiome, highlighted by the alpha diversity analysis, was further investigated in terms of beta diversity. The Non-metric multidimensional scaling (NMDS) of the samples (Bray Curtis metric) using only the chemical parameters showed a separation of springs categorized as different environments (Figure 4A). Springs that are used by the same facilities clustered together as well as those used for similar purposes. Spa springs were mostly located on the left side of the multivariate plot with some exceptions, belonging to the springs 1,111 and 7,359 (Levico and Fassa Terme, respectively). When the same type of plot was drawn starting from sequencing data (Figure 4B) we found a clustering pattern among the springs which was similar to the one obtained by the chemical data. Pearson’s correlation analysis between pairwise distances calculated using the chemical data and the ones calculated with the microbiological ones resulted in a correlation score of 0.52. Confirming that microbiological and chemical data were consistent in differentiating the springs.

### The analysis of shared ASVs reveals three distinct groups of springs

Focusing on the NMDS obtained using chemical metadata, a clear separation of the group of springs below −0.2 in NMDS 1 was observed. The group of samples belonging to the springs categorized as Spa showed different chemical properties from the other samples, so they were labelled as ST, while the remaining ones were labelled as TP.

**Figure 5:**
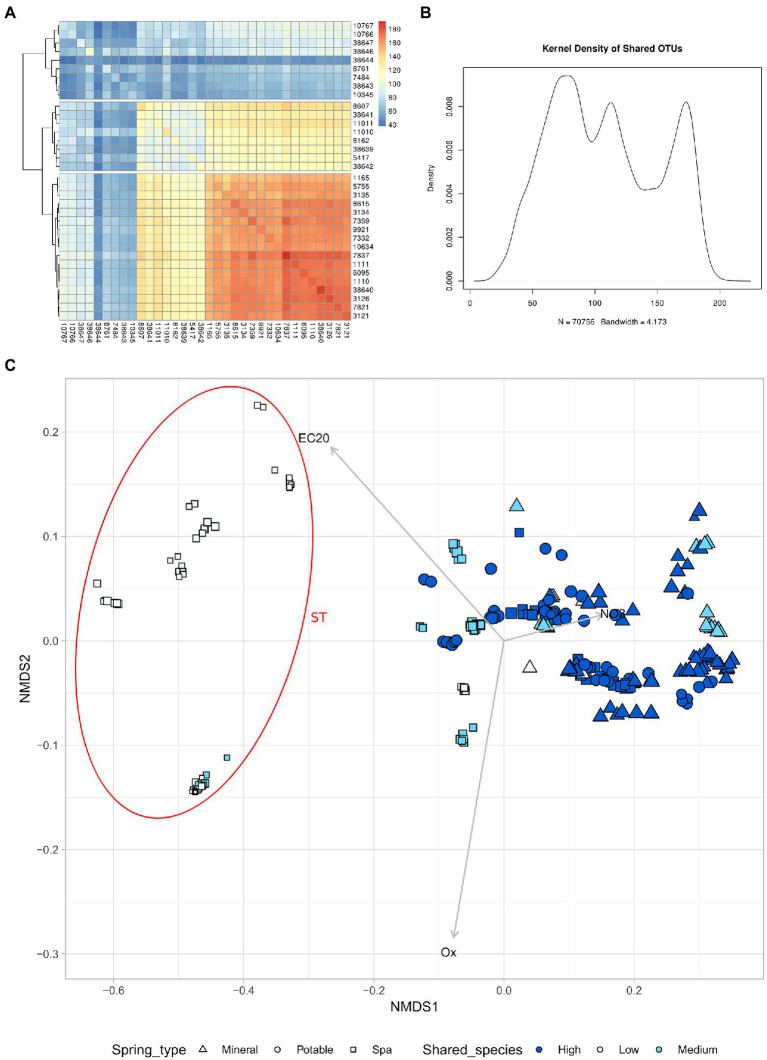
High nitrates concentration leads to a higher alpha diversity and more shared species. **(A)** Heatmap of the number of shared species between different springs (mean of all timepoints) **(B)** Kernel density of shared ASV **(C)** NMDS of all samples based on chemical parameters, the size of the point is proportional to the alpha diversity of the sample. The samples have been divided in 3 groups based on the number of shared species with other springs. High number of shared species (High), Medium number of shared species (Medium), low number of shared species (Low). Samples with NMDS1 lower than −0.2 have been assigned to the group ST.

The similarity between microbiomes in springs was investigated in relation to the number of shared ASVs between different samples. Considering the average number of ASV shared between two springs among all sampling timepoints, it was possible to distinguish 3 groups of springs: high number of shared ASVs (High), medium number of shared ASVs (Mid), and low number of shared ASVs (Low; Figure 5). This revealed that the majority of the samples that were linked to the ST cluster belonged to the “Low” group, which is also composed of springs with lower Alpha diversity.

### TP and ST groups showed distinct taxonomic compositions at the phylum level

The community composition of the springs was dominated by uncharacterized bacterial taxa. The 5 most represented ASV (g__Candidatus_Omnitrophus, g__Leptospirillum, g__Omnitrophales, c__Oligoflexia g__0319-6G20, p__Myxococcota g__bacteriap25) were present in all sequenced samples and represented on average more than 1% of the total number of ASVs, with maximum values above 6%. Just the Candidatus Omnitrophus (phylum Verrucomicrobiota), represented on average 6.8% of the profile reaching a maximum of 30.1%. The amount of uncharacterized ASVs increased at deeper taxonomic levels.

**Figure 6:**
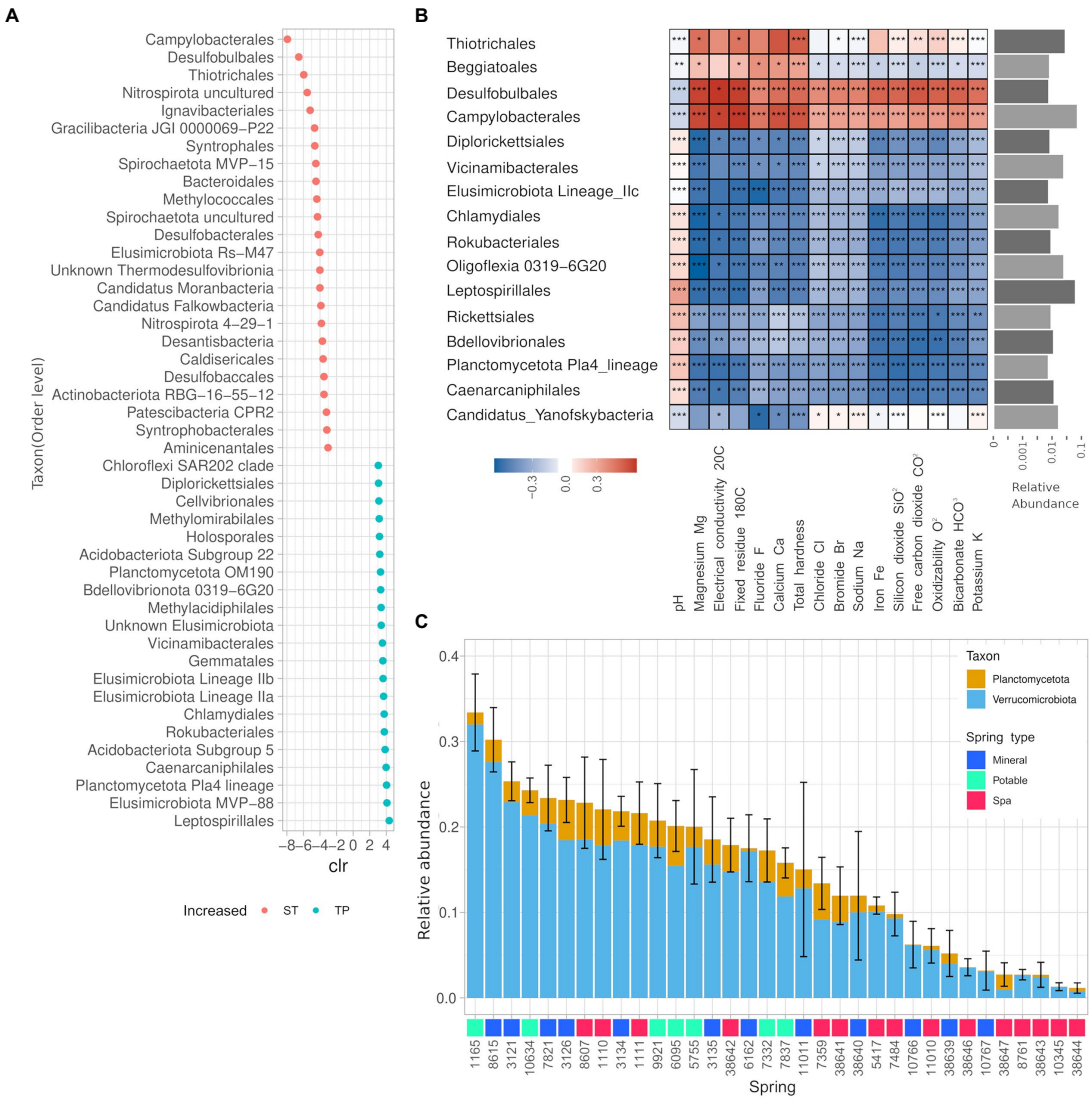
The microbial communities of the springs are largely uncharacterized and partially influenced by the physico-chemical properties of the water. **(A)** Summary of the ANCOM analysis on ST and TP groups, only the taxa with|clr| > 3. **(B)** Heatmap of the partial correlations for the presence of different taxons against chemical parameters of spring water. The taxa displayed showed a Spearman correlation score bigger than 0.5 for at least one of the measured chemical entities and an average relative abundance greater than 0.1%. **(C)** Average relative abundance of phyla belonging to the PVC superphylum for each spring over 2 years. Error bar relative to the sum of all phyla.

The ANCOM analysis for differentially abundant phyla revealed that the phyla Caldiserica and Lentisphaerae, and the CPR OP8, SR1, OP9, TPD-58 were enriched in the springs belonging to the ST group, while in the group TP Verrucomicrobia, Elusimicrobia, Planctomycetes, and Chlamydiae are present with significantly higher abundance. The analysis also found 391 differentially abundant genera, 44 of them (11%) belonging to the PVC superphylum (25 Verrucomicrobiota, 19 Planctomycetota). Thirty-five out of 44 of such genera were significantly enriched in the springs belonging to the TP group. The taxa displaying the highest centered-log ratio (clr) for the springs in the ST group were linked to the hydrocarbon degradation and sulfur cycle, namely Desulfobulbales, Campylobacterales, and Thiotrichales, whereas the taxa with higher clr for the springs from the TP group were Leptospirillales, Elusimicrobiota MVP-88, and Planctomycetota Pla4 (Figure 6A). The influence of the environmental parameters on single taxa has been evaluated using a Spearman partial correlation score corrected for nitrogen concentration. This analysis showed that only a fraction of the taxa is sensitive to the changes in the water chemical composition (Figure 6B). More precisely, the 14% of the detected genera showed a correlation score bigger than 0.5 for at least one of the measured chemical entities, and all the calculated scores remained in the range +/−0,7 indicating moderate correlations. A more thorough analysis of the relative abundances of the Phyla Planctomycetes and Verrucomicrobia showed a heterogeneous presence of those two taxa among all springs ranging from 2 to 30% of relative abundance, mostly represented by the Verrucomicrobia phylum (Figure 6C). The average abundance of Planctomycetes and Verrucomicrobia was found to be significantly lower in Spa Spring than in springs used for other purposes (Supplementary Figure 2).

## Discussion

Groundwater accounts for about a third of all freshwater storage on earth (Famiglietti, 2014), and they are among the most vulnerable sites to climate change (Taylor et al., 2013). In this environment, microbial communities are among the key players in biogeochemical cycles (Bell et al., 2018), water cleaning by removal of xenobiotic compounds (Payne and Floyd, 1990; Mikkonen et al., 2018; Vera et al., 2022), and primary production (Jewell et al., 2016). Due to the absence of light, photoautotrophy is impossible and the primary production is mainly performed by chemolithoautotrophs (Jewell et al., 2016). Most of the microbial biomass, however, is constituted by heterotrophs which are adapted to nutrient-poor environments (Griebler et al., 2014). Despite the increasing number of studies on groundwater microbiome, there are still open questions regarding the general patterns and processes shaping microbial diversity in such environments. While there is a general consensus on the fact that groundwater environments are quite stable in terms of physico-chemical parameters (Griebler and Lueders, 2009), flora and fauna (Cantonati et al., 2006), the composition of microbial communities seems to vary according to several parameters such as EC, discharge (Yan et al., 2021), and temperature and pH (Power et al., 2018). A number of studies suggest that the microbiome can be used as a tracer for water quality in such environments, microbiological tracers have been effective in detecting hydraulic interconnection (Rizzo et al., 2020), seawater intrusion (Hernàndez-Diaz et al., 2019), and seismic events in SPA settings (Valeriani et al., 2020). Therefore the knowledge of spatial and temporal community dynamics is fundamental for both conservation issues and for the management of the ecosystem services provided by the water springs. Most studies, however, focused on a relatively small scale, describing the microbial diversity in springs along a transect or within a single hydrographic basin (e.g., Pedron et al., 2019; Yan et al., 2020; Çelik and Keskin, 2022). The studies that focused on a large scale are indeed very few (e.g., Power et al., 2018; Li and Ma, 2019; Yan et al., 2021), and the ones accounting also for temporal variation are even less. The setting of the present study is constituted by a heterogeneous geological background, the 37 springs are evenly distributed over 13.000 km^2^, the time span covers two consecutive years with four yearly samples, and the water usage spans three categories. Power et al. (2018), performed a similar study on an area of 8.000 km^2^ for 21 months, and found a non-linear response of microbial diversity patterns to pH and temperature (Power et al., 2018). The present study did not witness this pattern, but probably because the ranges of pH and temperature values were narrower. In our dataset, spring diversity varies according to the season, i.e., the alpha diversity displays a cyclical variation being lowest in winter and highest in spring. However, the variation of alpha diversity, highlighted by the coefficient of variation of the Shannon index (Figure 1A), was not homogeneous across all springs. The variation of the diversity values within a spring could be due on one hand to the presence of different microniches and changing gradients through time, and on the other hand to the input of percolating water carrying microbial biomass. The latter gains more importance in shallow aquifers, where percolating water is more likely to carry a high number of alive bacterial cells. Indeed, Hubalek et al., 2016 found that shallow aquifers display larger variation in their diversity (Hubalek et al., 2016). Furthermore, increasing depth may result in a depletion in nutrients, which therefore sustain less diverse communities. Yet the microbiome of single springs showed less variation when compared to the variation of all springs in a single season.

Another interesting biogeographical pattern that was found in the present study is the presence of three main groups in the analysis of shared ASV (Figures 5A,B). This means that the springs could be classified according to the number of shared ASV into three categories, two of which (high and mid) showed a higher similarity at the beta-diversity analysis (Figure 5C). The springs belonging to the “low number of shared ASV” category were in great majority springs used for SPA, they tend to form a distinct cluster on the first axes (which correlates with the nitrate content), and they have a lower alpha diversity. The two clusters of samples, named “TP” and “ST,” host taxonomically distinct communities, being the former more enriched in taxa belonging to the Planctomycetes and- Verrucomicrobia phyla and the latter more rich of Caldiserica, Lentisphaera, and CPR phyla.

Taken together, the results reflect the characteristics of insularity of water springs (Reiss et al., 2016), resulting from the low connectivity among them, that favours the establishment of very peculiar microbial communities. According to Savio et al., 2018, alpine water springs microbial communities are constituted of two main components: the “autochthonous” microbes and the “transient” ones (Savio et al., 2018). The relative proportions of those two components may vary according to the catchment and the hydrogeology of the settings. However, in that paper, the authors could not speculate further about the origin of the autochthonous microbiome and the mechanisms of community assembly. We found that there are groups of springs sharing a number of ASVs distinct from the others, suggesting that in each spring there are specific environmental filters shaping the *in situ* microbial communities.

Regarding the use of spring water for human use, the consumption of bottled water has risen in the last decades (Mitache et al., 2019). This is due to the misbelief that bottled water is “microbiologically pure,” whereas it would be more appropriate to include a statement such as “microbiologically safe.” Indeed, recent studies support the hypothesis that gut microbiome composition may vary in association with the consumption of waters harboring different microbiomes (e.g., Dias et al., 2018; Barnich et al., 2021; Vanhaecke et al., 2021; Lugli et al., 2022). Indeed, Dias et al., 2018 showed that the mouse gut microbiome (both faecal and mucosa-adhered), changed between mice fed with water from different origins (Dias et al., 2018). Lugli et al., 2022 instead, demonstrated that the gut microbiome of a subject, who daily consumed tap water in the past 3 years, contained DNA sequences homologous to the genome of an unknown species of the *Curvibacter* genus found in the tap water itself (Lugli et al., 2022), supporting the hypothesis that there was horizontal transmission and colonization of the gut microbiome by this species. Similarly, the usage of SPA waters could affect the composition of the human skin microbiome, as it has been shown previously for ocean waters (Nielsen and Jiang, 2019), in patients with psoriasis after treatment at the La Roche-Posay Thermal Care Centre (Martin et al., 2015) and treatment in the dead sea (Brandwein et al., 2019). The effect could be even stronger in this case as the human skin microbiome has been shown to be among the least diverse among the mammalians (Ross et al., 2018). Thus the knowledge of the taxonomic composition of water microbiome acquires additional relevance in quality control of water for human consumption. This aspect could be explored also in agricultural settings, since most of the irrigation water derives from groundwater aquifers (Foster and Custodio, 2019; Riedel and Weber, 2020), yet it is unknown how water microbiome shapes the rhizosphere one.

The taxonomic profiles of the sampled spring’s microbiomes include many species which are known from previous studies to thrive in groundwater environments. Among the most prevalent genera, there were two genera belonging to the candidate phylum Omnitrophica (namely g__Candidatus_Omnitrophus and g__Omnitrophales). This taxon has been found in several environments including aquifers (Glöckner et al., 2010). Metagenome-Assembled Genomes affiliated to this phylum have been deeply characterized in a recent study on the metagenome of an Antarctic lake, which suggested that those taxa have a strong protein degradative capacity, and they are considered a obligate fermentative heterotroph which are deemed to establish metabolic interactions with Desulfobacterota (Williams et al., 2021). Members of the Oligoflexia and Myxococcota are also among the most represented ASVs, those phyla were recently established as new phylum replacing a subset of taxa within the phylum formerly known as Delta-Proteobacteria (Waite et al., 2020). Both phyla host species with predatory lifestyle, but with different predatory strategies, their role can be relevant in microbial food-web and organic matter turn-over, and it could have been overlooked in previous studies (e.g., Karwautz et al., 2022). The widespread presence of the iron-oxidizing genus Leptospirillum was consistent with previous findings in other similar studies (Farnleitner et al., 2005; Kostanjšek et al., 2013; Savio et al., 2019).

We found significant correlations between specific taxa and chemical parameters or spring type, also accounting for the autocorrelation of such parameters with nitrogen concentration. For example, the lineages Elusimicrobia IIa and IIb were found to be biomarkers for the springs “ST” (Figure 6A), whereas the Elusimicrobia lineage IIc displayed negative and significant correlation with most of the environmental parameters, suggesting an adaptation to an extremely oligotrophic environment (Figure 6B). The Elusimicrobia lineages we found are widespread in the environment (i.e., non-host-associated) and according to a recent comparative genomic study on this phylum, they are pervasive in groundwater environments (Méheust et al., 2020). The genome of groundwater Elusimicrobia encodes genes spanning a multitude of functions, including genes involved in nitrogen cycle and carbohydrate fermentation. Springs belonging to the “ST” category were characterized also by the presence of bacterial taxa involved in sulfur oxidation and reduction in aquatic, aphotic, microaerophilic/anoxic environments. More specifically Campylobacterales, Thiotrichales, and Desulfobulbales. Those taxa also display significant correlations with the environmental parameters (negative with pH and positive with most solute). The genus Gallionella contains chemolithotrophic species adapted to low-oxygen conditions (Hallbeck and Pedersen, 1991), this genus correlates positively with the iron concentration, and presents a negative correlation with pH (Supplementary Figure 3), which is coherent with a possible iron oxidation activity.

We found a widespread distribution, at relatively high abundance, of ASV affiliated to the Candidate Phyla Radiation (CPR) or to the phyla Planctomycetes and Verrucomicrobia PVC superphyla in the water for human consumption. Such bacteria are highly adapted to the oligotrophic conditions encountered in the groundwater environments, which result in low metabolic rate and shrunken cells (Jørgensen, 2012). Such phyla are made of mostly uncultured species which are not detected by standard quality controls, but are harmless anyway because, in copiotrophic environments such as the host-associated ones, they are easily outcompeted by the species belonging to the native microbiome. While Planctomycetes is a well-known phylum in this type of environment, and it is in agreement with at least another study focusing on groundwater for human consumption in the Netherlands (Roeselers et al., 2015), the reports regarding Verrucomicrobia in such environments are less frequent, species belonging to this phylum are deemed to be involved in the degradation of (poly)saccharide compounds derived probably by terrestrial environments above the groundwater (He et al., 2017). Another unexpected finding regarded Chlamydiales, which were found to be enriched in TP springs (Figure 6A), such phylum hosts many obligate intracellular species, probably symbionts of the protozoan stygofauna. A great diversity of Chlamydia was found in the deep ocean sediments (Dharamshi et al., 2020), our study suggests a similar situation also in groundwater. Lastly, Savio et al., 2019 observed that Flavobacteriia abundance fluctuations were strictly bound to discharge events (Savio et al., 2019). In our experiment, the discharge was not measured during the sampling, so we used the season as a proxy for the discharge; however, we did not witness any significant correlation between this parameter and the abundance of Flavobacteria.

## Conclusion

The recent discoveries on the effect of water microbiome on the human one point toward the need of a thorough characterization of the microbiological quality of the waters for human consumption. The concept of using bacteria as tracers of water quality is already applied in the standard quality check, but recent methodological advances allow to get a more complete overview of the microbiome as a whole instead of specific taxa, thus increasing the resolution of the analysis. We provided proof that the emerging properties of the microbiome provide accurate and replicable information about spring water quality in Trentino region over 2 years of sampling. We found that spring diversity fluctuates through time, and this fluctuation does not have the same magnitude in all springs. Indeed some springs are more stable than others, and the coefficient of variation of the Shannon diversity may vary up to one order of magnitude. Furthermore, we found that the microbiome composition of each spring tends to remain more similar to itself through time than to other springs within the same season, suggesting stronger niche selection in the area of study. At last, we found that Planctomycetes and Verrucomicrobia were associated with the water usage, being represented at higher abundance in the springs used for mineral or potable purposes. Our results sum up to the recent literature in support of the use of microbial communities as bioindicators for groundwater quality assessment (e.g., Smith et al., 2015; Voisin et al., 2016; Fillinger et al., 2019; Korbel et al., 2022), and set the stage for future, more extensive characterization of water springs microbial communities, which can be used by the stakeholders and the decision makers to improve the conservation, exploitation and management plans for this important resource.

## Data availability statement

The datasets presented in this study can be found in online repositories. The names of the repository/repositories and accession number(s) can be found at: https://www.ncbi.nlm.nih.gov/, PRJNA837552.

## Author contributions

OJ and MC jointly designed the study. RP and WC isolated the samples. RP, AE, and OJ wrote the manuscript. All authors analyzed and interpreted the data, and have read and approved the final version of the manuscript.

## Conflict of interest

Part of the results presented herein are derived from the Master thesis of WC at the University of Trento, Italy. This represents the only medium these results have appeared in, and is in line with the author’s university policy. The thesis can be accessed online at https://webapps.unitn.it/Biblioteca/en/Web/Tesi.

The remaining authors declare that the research was conducted in the absence of any commercial or financial relationships that could be construed as a potential conflict of interest.

## Publisher’s note

All claims expressed in this article are solely those of the authors and do not necessarily represent those of their affiliated organizations, or those of the publisher, the editors and the reviewers. Any product that may be evaluated in this article, or claim that may be made by its manufacturer, is not guaranteed or endorsed by the publisher.

## References

[ref1] Aburto-Medina A. Shahsavari E. Cohen M. Mantri N. Ball A. S. (2020). Analysis of the microbiome (bathing biome) in geothermal waters from an Australian balneotherapy Centre. Water 12:1705. doi: 10.3390/W12061705

[ref2] Andrews S. (2010). FastQC: A quality control tool for high throughput sequence data. Available at: http://www.bioinformatics.babraham.ac.uk/projects/

[ref3] Barnich N. Rodrigues M. Sauvanet P. Chevarin C. Denis S. Le Goff O.. (2021). Beneficial effects of natural mineral waters on intestinal inflammation and the mucosa-associated microbiota. Int. J. Mol. Sci. 22:4336. doi: 10.3390/ijms22094336, PMID: 33919372 PMC8122343

[ref4] Bell E. Lamminmäki T. Alneberg J. Andersson A. F. Qian C. Xiong W.. (2018). Biogeochemical cycling by a low-diversity microbial community in deep groundwater. Front. Microbiol. 9:2129. doi: 10.3389/fmicb.2018.02129, PMID: 30245678 PMC6137086

[ref5] Bolyen E. Rideout J. R. Dillon M. R. Bokulich N. A. Abnet C. C. Al-Ghalith G. A.. (2019). Reproducible, interactive, scalable and extensible microbiome data science using QIIME 2. Nat. Biotechnol. 37, 852–857. doi: 10.1038/s41587-019-0209-9, PMID: 31341288 PMC7015180

[ref6] Bourrain M. Suzuki M. T. Calvez A. West N. J. Lions J. Lebaron P. (2020). In-depth prospection of Avène thermal spring water reveals an uncommon and stable microbial community. J. Eur. Acad. Dermatol. Venereol. 34, 8–14. doi: 10.1111/jdv.16599, PMID: 32870559

[ref7] Brandwein M. Fuks G. Israel A. Sabbah F. Hodak E. Szitenberg A.. (2019). Skin microbiome compositional changes in atopic dermatitis accompany Dead Sea Climatotherapy. Photochem. Photobiol. 95, 1446–1453. doi: 10.1111/php.13119, PMID: 31074874

[ref8] Bruno A. Sandionigi A. Bernasconi M. Panio A. Labra M. Casiraghi M. (2018). Changes in the drinking water microbiome: effects of water treatments along the flow of two drinking water treatment plants in a urbanized area, Milan (Italy). Front. Microbiol. 9:2557. doi: 10.3389/fmicb.2018.02557, PMID: 30429832 PMC6220058

[ref9] Bruno A. Sandionigi A. Rizzi E. Bernasconi M. Vicario S. Galimberti A.. (2017). Exploring the under-investigated “microbial dark matter” of drinking water treatment plants. Sci. Rep. 7:44350. doi: 10.1038/srep44350, PMID: 28290543 PMC5349567

[ref10] Burri N. M. Weatherl R. Moeck C. Schirmer M. (2019). A review of threats to groundwater quality in the anthropocene. Sci. Total Environ. 684, 136–154. doi: 10.1016/j.scitotenv.2019.05.236, PMID: 31153063

[ref11] Callahan B. J. McMurdie P. J. Rosen M. J. Han A. W. Johnson A. J. A. Holmes S. P. (2016). DADA2: high-resolution sample inference from Illumina amplicon data. Nat. Methods 13, 581–583. doi: 10.1038/nmeth.3869, PMID: 27214047 PMC4927377

[ref12] Cantonati M. Gerecke R. Bertuzzi E. (2006). Springs of the Alps – sensitive ecosystems to environmental change: from biodiversity assessments to long-term studies. Hydrobiologia 562, 59–96. doi: 10.1007/s10750-005-1806-9

[ref13] Caporaso J. G. Lauber C. L. Walters W. A. Berg-Lyons D. Huntley J. Fierer N.. (2012). Ultra-high-throughput microbial community analysis on the Illumina HiSeq and MiSeq platforms. ISME J. 6, 1621–1624. doi: 10.1038/ismej.2012.8, PMID: 22402401 PMC3400413

[ref14] Çelik I. Keskin E. (2022). Revealing the microbiome of four different thermal springs in Turkey with environmental DNA Metabarcoding. Biology 11:998. doi: 10.3390/biology11070998 36101376 PMC9311576

[ref15] Dharamshi J. E. Tamarit D. Eme L. Stairs C. W. Martijn J. Homa F.. (2020). Marine sediments illuminate Chlamydiae diversity and evolution. Curr. Biol. 30, 1032–1048.e7. doi: 10.1016/j.cub.2020.02.016, PMID: 32142706

[ref16] Dias M. F. Reis M. P. Acurcio L. B. Carmo A. O. Diamantino C. F. Motta A. M.. (2018). Changes in mouse gut bacterial community in response to different types of drinking water. Water Res. 132, 79–89. doi: 10.1016/j.watres.2017.12.052, PMID: 29306702

[ref17] Famiglietti J. S. (2014). The global groundwater crisis. Nat. Clim. Chang. 4, 945–948. doi: 10.1038/nclimate2425

[ref18] Farnleitner A. H. Wilhartitz I. Ryzinska G. Kirschner A. K. T. Stadler H. Burtscher M. M.. (2005). Bacterial dynamics in spring water of alpine karst aquifers indicates the presence of stable autochthonous microbial endokarst communities. Environ. Microbiol. 7, 1248–1259. doi: 10.1111/j.1462-2920.2005.00810.x, PMID: 16011762

[ref19] Fillinger L. Hug K. Trimbach A. M. Wang H. Kellermann C. Meyer A.. (2019). The D-A-(C) index: a practical approach towards the microbiological-ecological monitoring of groundwater ecosystems. Water Res. 163:114902. doi: 10.1016/j.watres.2019.114902, PMID: 31362215

[ref20] Foster S. Custodio E. (2019). Groundwater resources and intensive agriculture in Europe – can regulatory agencies cope with the threat to sustainability? Water Resour. Manag. 33, 2139–2151. doi: 10.1007/s11269-019-02235-6

[ref21] Giliberti R. Cavaliere S. Mauriello I. E. Ercolini D. Pasolli E. (2022). Host phenotype classification from human microbiome data is mainly driven by the presence of microbial taxa. PLoS Comput. Biol. 18, e1010066–e1010022. doi: 10.1371/journal.pcbi.1010066, PMID: 35446845 PMC9064115

[ref22] Glöckner J. Kube M. Shrestha P. M. Weber M. Glöckner F. O. Reinhardt R.. (2010). Phylogenetic diversity and metagenomics of candidate division OP3. Environ. Microbiol. 12, 1218–1229. doi: 10.1111/j.1462-2920.2010.02164.x, PMID: 20158507

[ref23] Griebler C. Avramov M. (2015). Groundwater ecosystem services: a review. Freshw. Sci. 34, 355–367. doi: 10.1086/679903

[ref24] Griebler C. Lueders T. (2009). Microbial biodiversity in groundwater ecosystems. Freshw. Biol. 54, 649–677. doi: 10.1111/j.1365-2427.2008.02013.x

[ref25] Griebler C. Malard F. Lefébure T. (2014). Current developments in groundwater ecology-from biodiversity to ecosystem function and services. Curr. Opin. Biotechnol. 27, 159–167. doi: 10.1016/j.copbio.2014.01.018, PMID: 24590188

[ref26] Hallbeck L. Pedersen K. (1991). Autotrophic and mixotrophic growth of *Gallionella ferruginea*. J. Gen. Microbiol. 137, 2657–2661. doi: 10.1099/00221287-137-11-2657

[ref27] He S. Stevens S. L. R. Chan L.-K. Bertilsson S. Glavina del Rio T. Tringe S. G.. (2017). Ecophysiology of freshwater *Verrucomicrobia* inferred from Metagenome-assembled genomes. mSphere 2:e00277-17. doi: 10.1128/msphere.00277-17, PMID: 28959738 PMC5615132

[ref28] Hernàndez-Diaz R. Petrella E. Bucci A. Naclerio G. Feo A. Sferra G.. (2019). Integrating hydrogeological and microbiological data and modelling to characterize the hydraulic features and behaviour of coastal carbonate aquifers: a case in western Cuba. Water 11:1989. doi: 10.3390/w11101989

[ref29] Herrmann M. Rusznyák A. Akob D. M. Schulze I. Opitz S. Totsche K. U.. (2015). Large fractions of CO2-fixing microorganisms in pristine limestone aquifers appear to be involved in the oxidation of reduced sulfur and nitrogen compounds. Appl. Environ. Microbiol. 81, 2384–2394. doi: 10.1128/AEM.03269-14, PMID: 25616797 PMC4357952

[ref30] Hubalek V. Wu X. Eiler A. Buck M. Heim C. Dopson M.. (2016). Connectivity to the surface determines diversity patterns in subsurface aquifers of the Fennoscandian shield. ISME J. 10, 2447–2458. doi: 10.1038/ismej.2016.36, PMID: 27022994 PMC5030689

[ref31] Hull N. M. Ling F. Pinto A. J. Albertsen M. Jang H. G. Hong P. Y.. (2019). Drinking water microbiome project: is it time? Trends Microbiol. 27, 670–677. doi: 10.1016/j.tim.2019.03.011, PMID: 31031092

[ref32] Jewell T. N. M. Karaoz U. Brodie E. L. Williams K. H. Beller H. R. (2016). Metatranscriptomic evidence of pervasive and diverse chemolithoautotrophy relevant to C, S, N and Fe cycling in a shallow alluvial aquifer. ISME J. 10, 2106–2117. doi: 10.1038/ismej.2016.25, PMID: 26943628 PMC4989316

[ref33] Jørgensen B. B. (2012). Shrinking majority of the deep biosphere. Proc. Natl. Acad. Sci. U. S. A. 109, 15976–15977. doi: 10.1073/pnas.1213639109, PMID: 23012471 PMC3479554

[ref34] Karwautz C. Zhou Y. Kerros M.-E. Weinbauer M. G. Griebler C. (2022). Bottom-up control of the groundwater microbial food-web in an alpine aquifer. Front. Ecol. Evol. 10, 1–14. doi: 10.3389/fevo.2022.854228

[ref35] Kolvenbach B. A. Helbling D. E. Kohler H. P. E. Corvini P. F. X. (2014). Emerging chemicals and the evolution of biodegradation capacities and pathways in bacteria. Curr. Opin. Biotechnol. 27, 8–14. doi: 10.1016/j.copbio.2013.08.017, PMID: 24863891

[ref36] Korbel K. L. Rutlidge H. Hose G. C. Eberhard S. M. Andersen M. S. (2022). Dynamics of microbiotic patterns reveal surface water groundwater interactions in intermittent and perennial streams. Sci. Total Environ. 811:152380. doi: 10.1016/j.scitotenv.2021.152380, PMID: 34914978

[ref37] Kostanjšek R. Pašić L. Daims H. Sket B. (2013). Structure and community composition of sprout-like bacterial aggregates in a Dinaric karst subterranean stream. Microb. Ecol. 66, 5–18. doi: 10.1007/s00248-012-0172-1, PMID: 23314097

[ref38] Kumar S. Herrmann M. Thamdrup B. Schwab V. F. Geesink P. Trumbore S. E.. (2017). Nitrogen loss from pristine carbonate-rock aquifers of the hainich critical zone exploratory (Germany) is primarily driven by chemolithoautotrophic anammox processes. Front. Microbiol. 8:1951. doi: 10.3389/fmicb.2017.01951, PMID: 29067012 PMC5641322

[ref39] Li L. Ma Z. (2019). Global microbiome diversity scaling in hot springs with DAR (diversity-area relationship) profiles. Front. Microbiol. 10:118. doi: 10.3389/fmicb.2019.00118, PMID: 30853941 PMC6395440

[ref40] Lozupone C. Lladser M. E. Knights D. Stombaugh J. Knight R. (2011). UniFrac: an effective distance metric for microbial community comparison. ISME J. 5, 169–172. doi: 10.1038/ismej.2010.133, PMID: 20827291 PMC3105689

[ref41] Lugli G. A. Longhi G. Mancabelli L. Alessandri G. Tarracchini C. Fontana F.. (2022). Tap water as a natural vehicle for microorganisms shaping the human gut microbiome. Environ. Microbiol. doi: 10.1111/1462-2920.15988, PMID: 35355372 PMC9790288

[ref42] Martin R. Henley J. B. Sarrazin P. Seité S. (2015). Skin microbiome in patients with psoriasis before and after balneotherapy at the thermal care center of la roche-posay. J. Drugs Dermatol. 74:AB276. doi: 10.1016/j.jaad.2016.02.1062 26659932

[ref43] Méheust R. Castelle C. J. Matheus Carnevali P. B. Farag I. F. He C. Chen L. X.. (2020). Groundwater Elusimicrobia are metabolically diverse compared to gut microbiome Elusimicrobia and some have a novel nitrogenase paralog. ISME J. 14, 2907–2922. doi: 10.1038/s41396-020-0716-1, PMID: 32681159 PMC7785019

[ref44] Mikkonen A. Yläranta K. Tiirola M. Dutra L. A. L. Salmi P. Romantschuk M.. (2018). Successful aerobic bioremediation of groundwater contaminated with higher chlorinated phenols by indigenous degrader bacteria. Water Res. 138, 118–128. doi: 10.1016/j.watres.2018.03.033, PMID: 29574199

[ref45] Miller J. D. Workman C. L. Panchang S. V. Sneegas G. Adams E. A. Young S. L.. (2021). Water security and nutrition: current knowledge and research opportunities. Adv. Nutr. 12, 2525–2539. doi: 10.1093/advances/nmab075, PMID: 34265039 PMC8634318

[ref46] Mirarab S. Nguyen N. Warnow T. (2012). SEPP: SATé-enabled phylogenetic placement. Pac. Symp. Biocomput. 247–258 doi: 10.1142/9789814366496_0024, . 22174280

[ref47] Mitache M. M. Neagu L. Curutiu C. Holban A. Iordache E.-G. P. Chifiriuc M. C. (2019). Microbiological and chemical characterization of bottled waters. Bottl. Packag. Water 4, 209–226. doi: 10.1016/b978-0-12-815272-0.00008-8

[ref48] Nielsen M. C. Jiang S. C. (2019). Alterations of the human skin microbiome after ocean water exposure. Mar. Pollut. Bull. 145, 595–603. doi: 10.1016/j.marpolbul.2019.06.047, PMID: 31590829 PMC8061468

[ref49] Nowak M. E. Schwab V. F. Lazar C. S. Behrendt T. Kohlhepp B. Totsche K. U.. (2017). Carbon isotopes of dissolved inorganic carbon reflect utilization of different carbon sources by microbial communities in two limestone aquifer assemblages. Hydrol. Earth Syst. Sci. 21, 4283–4300. doi: 10.5194/hess-21-4283-2017

[ref50] Oksanen J. Blanchet F. G. Friendly M. Kindt R. Legendre P. Mcglinn D.. (2019). Vegan: community ecology package. R package version 2.4-2. *Community Ecol. Packag*. 2.5–6.

[ref51] Opitz S. Küsel K. Spott O. Totsche K. U. Herrmann M. (2014). Oxygen availability and distance to surface environments determine community composition and abundance of ammonia-oxidizing prokaroytes in two superimposed pristine limestone aquifers in the Hainich region, Germany. FEMS Microbiol. Ecol. 90, 39–53. doi: 10.1111/1574-6941.12370, PMID: 24953994

[ref52] Payne J. R. Floyd M. S. (1990). Petroleum and chlorinated hydrocarbon analysis in support of in vitro studies of natural anaerobic and aerobic microbial degradation of xenobiotics in contaminated groundwater and soil. Int. J. Environ. Anal. Chem. 39, 101–120. doi: 10.1080/03067319008027687

[ref53] Pedron R. Esposito A. Bianconi I. Pasolli E. Tett A. Asnicar F.. (2019). Genomic and metagenomic insights into the microbial community of a thermal spring. Microbiome 7:8. doi: 10.1186/s40168-019-0625-6, PMID: 30674352 PMC6343286

[ref54] Power J. F. Carere C. R. Lee C. K. Wakerley G. L. J. Evans D. W. Button M.. (2018). Microbial biogeography of 925 geothermal springs in New Zealand. Nat. Commun. 9:2876. doi: 10.1038/s41467-018-05020-y, PMID: 30038374 PMC6056493

[ref55] Quast C. Pruesse E. Yilmaz P. Gerken J. Schweer T. Yarza P.. (2013). The SILVA ribosomal RNA gene database project: improved data processing and web-based tools. Nucleic Acids Res. 41, D590–D596. doi: 10.1093/nar/gks1219 23193283 PMC3531112

[ref56] Reiss M. Martin P. Gerecke R. von Fumetti S. (2016). Limno-ecological characteristics and distribution patterns of spring habitats and invertebrates from the lowlands to the Alps. Environ. Earth Sci. 75:1033. doi: 10.1007/s12665-016-5818-8

[ref57] Riedel T. Weber T. K. D. (2020). Review: the influence of global change on Europe’s water cycle and groundwater recharge. Hydrogeol. J. 28, 1939–1959. doi: 10.1007/s10040-020-02165-3

[ref58] Rizzo P. Petrella E. Bucci A. Salvioli-Mariani E. Chelli A. Sanangelantoni A. M.. (2020). Studying hydraulic interconnections in low-permeability media by using bacterial communities as natural tracers. Water 12:1795. doi: 10.3390/w12061795

[ref59] Roeselers G. Coolen J. van der Wielen P. W. J. J. Jaspers M. C. Atsma A. de Graaf B.. (2015). Microbial biogeography of drinking water: patterns in phylogenetic diversity across space and time. Environ. Microbiol. 17, 2505–2514. doi: 10.1111/1462-2920.12739, PMID: 25581482

[ref60] Ross A. A. Müller K. M. Scott Weese J. Neufeld J. D. (2018). Comprehensive skin microbiome analysis reveals the uniqueness of human skin and evidence for phylosymbiosis within the class Mammalia. Proc. Natl. Acad. Sci. U. S. A. 115, E5786–E5795. doi: 10.1073/pnas.1801302115, PMID: 29871947 PMC6016819

[ref61] Roy C. Rameez M. J. Haldar P. K. Peketi A. Mondal N. Bakshi U.. (2020). Microbiome and ecology of a hot spring-microbialite system on the trans-Himalayan plateau. Sci. Rep. 10:5917. doi: 10.1038/s41598-020-62797-z, PMID: 32246033 PMC7125080

[ref62] Savio D. Stadler P. Reischer G. H. Demeter K. Linke R. B. Blaschke A. P.. (2019). Spring water of an alpine karst aquifer is dominated by a taxonomically stable but discharge-responsive bacterial community. Front. Microbiol. 10:28. doi: 10.3389/fmicb.2019.00028, PMID: 30828319 PMC6385617

[ref63] Savio D. Stadler P. Reischer G. H. Kirschner A. K. T. Demeter K. Linke R.. (2018). Opening the black box of spring water microbiology from alpine karst aquifers to support proactive drinking water resource management. WIREs Water 5:e1282. doi: 10.1002/wat2.1282, PMID: 29780584 PMC5947618

[ref64] Schwab V. F. Herrmann M. Roth V. N. Gleixner G. Lehmann R. Pohnert G.. (2017). Functional diversity of microbial communities in pristine aquifers inferred by PLFA- and sequencing-based approaches. Biogeosciences 14, 2697–2714. doi: 10.5194/bg-14-2697-2017

[ref65] Smith M. B. Rocha A. M. Smillie C. S. Olesen S. W. Paradis C. Wu L.. (2015). Natural bacterial communities serve as quantitative geochemical biosensors. MBio 6, e00326–e00315. doi: 10.1128/mBio.00326-15, PMID: 25968645 PMC4436078

[ref66] Taylor R. G. Scanlon B. Döll P. Rodell M. Van Beek R. Wada Y.. (2013). Ground water and climate change. Nat. Clim. Chang. 3, 322–329. doi: 10.1038/nclimate1744

[ref67] Valeriani F. Gianfranceschi G. Romano Spica V. (2020). The microbiota as a candidate biomarker for SPA pools and SPA thermal spring stability after seismic events. Environ. Int. 137:105595. doi: 10.1016/j.envint.2020.105595, PMID: 32106051

[ref68] Vanhaecke T. Bretin O. Poirel M. Tap J. (2021). Drinking water source and intake are associated with distinct gut microbiota signatures in US and UK populations. J. Nutr. 152, 171–182. doi: 10.1093/jn/nxab312, PMID: 34642755 PMC8754568

[ref69] Varga C. (2019). To treat or not to treat? Misbeliefs in spa water disinfection. Int. J. Biometeorol. 63, 1135–1138. doi: 10.1007/s00484-019-01722-0 31127425

[ref70] Vera A. Wilson F. P. Cupples A. M. (2022). Predicted functional genes for the biodegradation of xenobiotics in groundwater and sediment at two contaminated naval sites. Appl. Microbiol. Biotechnol. 106, 835–853. doi: 10.1007/s00253-021-11756-3, PMID: 35015144

[ref71] Voisin J. Cournoyer B. Mermillod-Blondin F. (2016). Assessment of artificial substrates for evaluating groundwater microbial quality. Ecol. Indic. 71, 577–586. doi: 10.1016/j.ecolind.2016.07.035

[ref72] Waite D. W. Chuvochina M. Pelikan C. Parks D. H. Yilmaz P. Wagner M.. (2020). Proposal to reclassify the proteobacterial classes deltaproteobacteria and oligoflexia, and the phylum thermodesulfobacteria into four phyla reflecting major functional capabilities. Int. J. Syst. Evol. Microbiol. 70, 5972–6016. doi: 10.1099/ijsem.0.004213, PMID: 33151140

[ref73] Williams T. J. Allen M. A. Berengut J. F. Cavicchioli R. (2021). Shedding light on microbial “dark matter”: insights into novel Cloacimonadota and Omnitrophota from an Antarctic Lake. Front. Microbiol. 12:2947. doi: 10.3389/fmicb.2021.741077, PMID: 34707591 PMC8542988

[ref74] Yan L. Hermans S. M. Totsche K. U. Lehmann R. Herrmann M. Küsel K. (2021). Groundwater bacterial communities evolve over time in response to recharge. Water Res. 201:117290. doi: 10.1016/j.watres.2021.117290, PMID: 34130083

[ref75] Yan L. Herrmann M. Kampe B. Lehmann R. Totsche K. U. Küsel K. (2020). Environmental selection shapes the formation of near-surface groundwater microbiomes. Water Res. 170:115341. doi: 10.1016/j.watres.2019.115341, PMID: 31790889

[ref76] Zhang Y. Oh S. Liu W. T. (2017). Impact of drinking water treatment and distribution on the microbiome continuum: an ecological disturbance’s perspective. Environ. Microbiol. 19, 3163–3174. doi: 10.1111/1462-2920.13800, PMID: 28654183

