# Supplementary material for: Microbiome characterization of alpine water springs for human consumption reveals site- and usage-specific microbial signatures
Source: Front Microbiol. 2022 Oct 5;13:946460. doi: 10.3389/fmicb.2022.946460 (PMC9581249; doi:10.3389/fmicb.2022.946460)
Supplement: Supplementary file 1 [file Table_1.DOCX]

**Supplementary table 1**: Summary of the sampled springs and outcome of the library preparation procedure and sequencing. Green slots represent successfully sequenced samples, red cells represent samples that failed DNA extraction or amplification during library preparation, yellow slots represent samples that had a successful sequencing but were excluded due to a read count lower than 30000.

| **Spring Code** | **Winter 2018** | **Spring 2018** | **Summer 2018** | **Autumn 2018** | **Winter 2019** | **Spring 2019** | **Summer 2019** | **Autumn 2019** |
| --- | --- | --- | --- | --- | --- | --- | --- | --- |
| **10345** |  |  |  |  |  |  |  |  |
| **10634** |  |  |  |  |  |  |  | Amp. fail |
| **10766** |  |  |  |  |  |  |  |  |
| **10767** |  |  |  |  |  |  |  |  |
| **11010** |  |  |  |  |  |  |  |  |
| **11011** |  |  |  |  |  |  |  |  |
| **1110** |  |  |  |  |  |  |  |  |
| **1111** |  |  |  |  |  |  |  |  |
| **1165** |  |  |  |  |  |  |  |  |
| **3121** |  |  |  |  |  |  |  |  |
| **3126** |  |  |  |  |  |  |  |  |
| **3134** |  |  |  |  |  |  |  |  |
| **3135** |  |  |  |  |  |  |  |  |
| **38639** | Amp. fail |  | Low reads |  |  |  |  |  |
| **38640** |  |  |  |  |  |  |  | Low reads |
| **38641** |  |  |  |  |  |  |  |  |
| **38642** |  |  |  |  |  |  |  |  |
| **38643** |  |  |  |  |  |  |  |  |
| **38644** |  |  |  |  |  |  |  |  |
| **38646** | Amp. fail |  |  |  |  |  |  |  |
| **38647** |  |  |  |  |  |  |  |  |
| **5417** |  |  |  |  |  |  |  |  |
| **5755** |  |  |  |  |  |  |  |  |
| **6095** |  |  |  |  |  |  |  |  |
| **6162** |  |  |  |  |  |  |  |  |
| **7332** |  |  |  |  |  |  |  |  |
| **7359** |  |  |  |  |  |  |  |  |
| **7484** |  |  |  |  |  |  |  |  |
| **7821** |  | Amp. fail |  |  |  |  |  |  |
| **7837** |  |  |  |  |  |  |  |  |
| **8607** |  |  |  |  |  |  |  |  |
| **8615** |  |  |  |  |  |  |  |  |
| **8761** |  |  |  |  |  |  |  |  |
| **9921** | Amp. fail |  |  |  |  |  |  |  |

**Supplementary table 2**: List of measured chemical parameters, method of analysis and guidelines followed for the analysis.


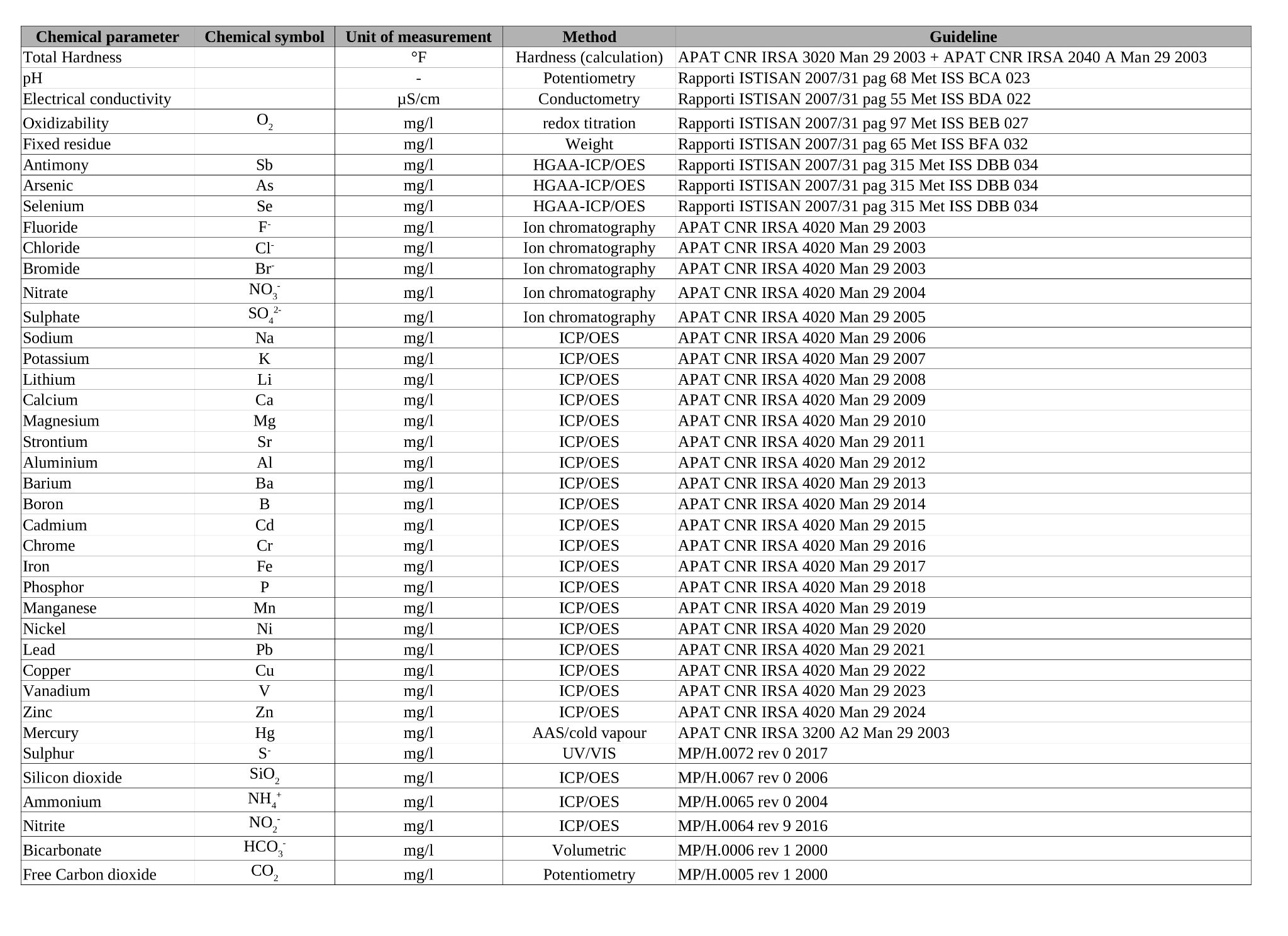


**Supplementary table 3**: Wilcoxon test result on shannon alpha diversity values for paired samples between seasons


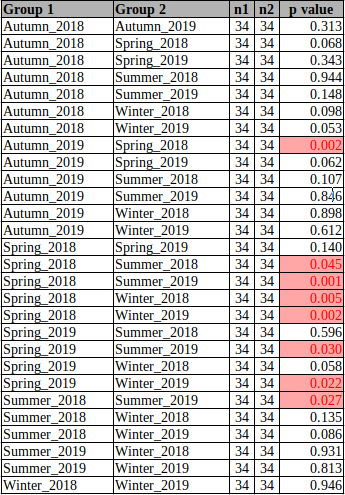


**
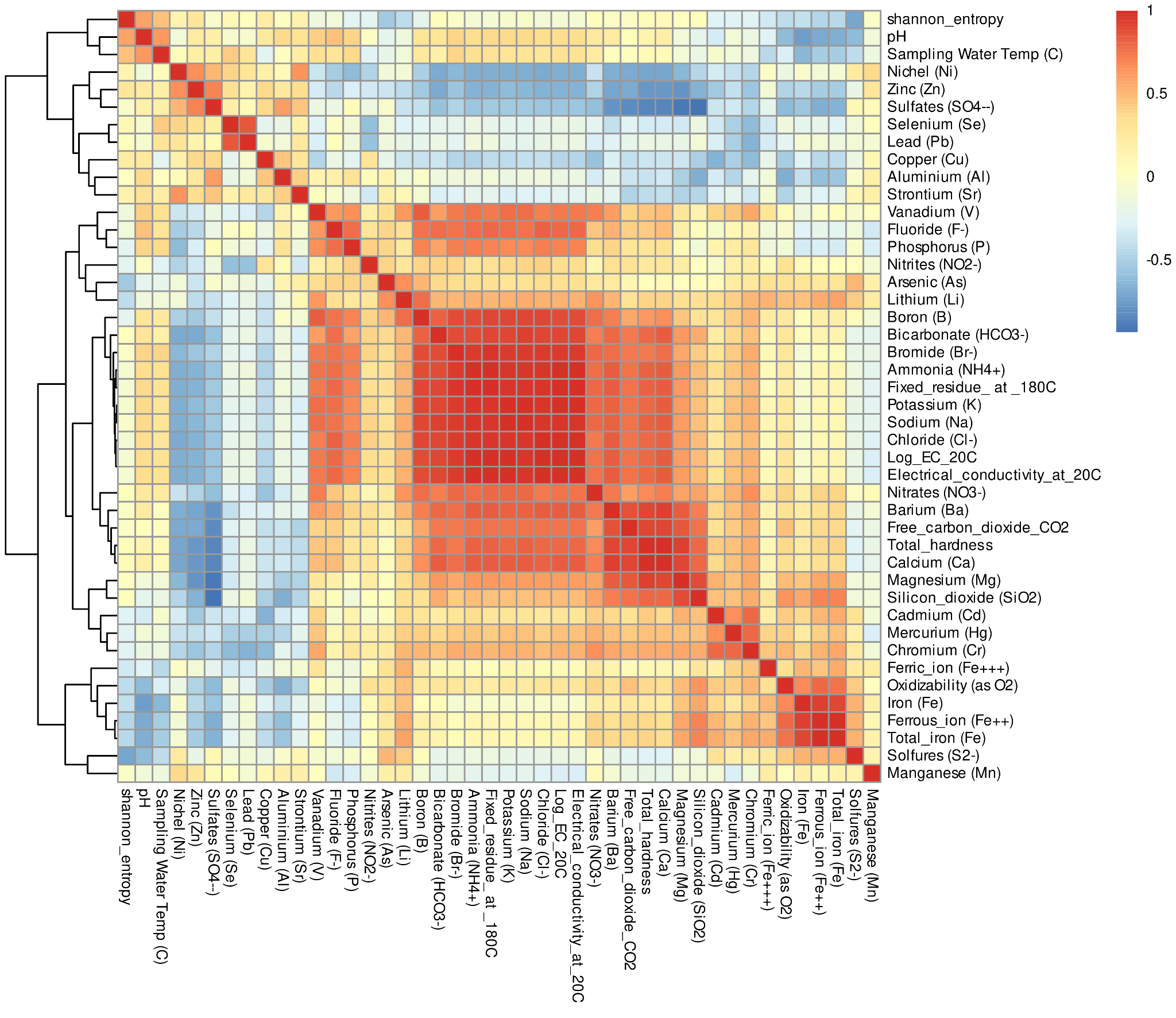
Supplementary Figure 1**. Reciprocal correlation analysis of chemical data using the Spearman correlation score.


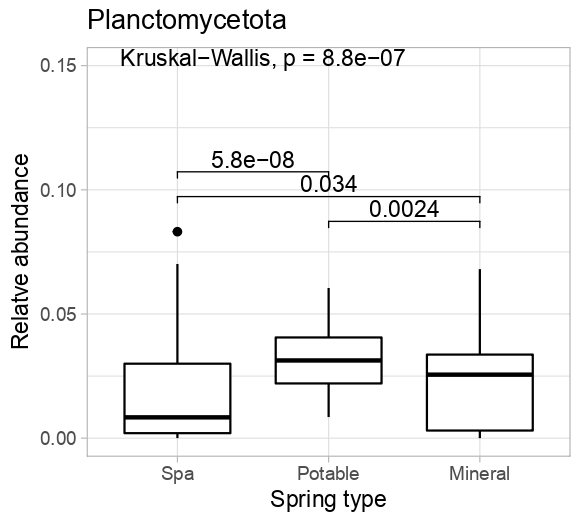

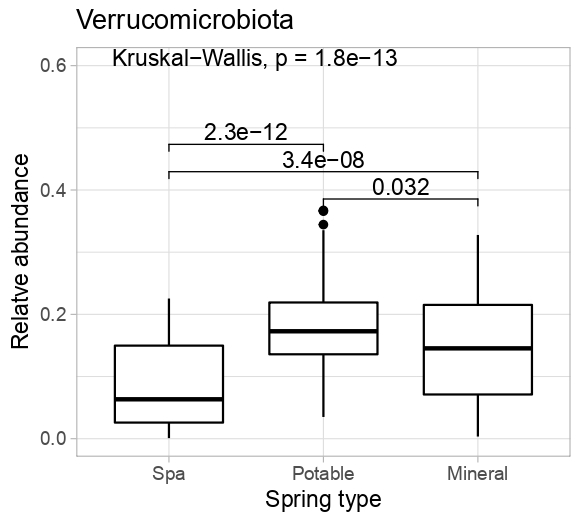


**Supplementary Figure 2**. Pairwise Wilcoxon test and Kruskal-Wallis test for the statistical analysis of the Planctomycetota and Verrucomicrobiota abundance in springs used for different purposes.

**
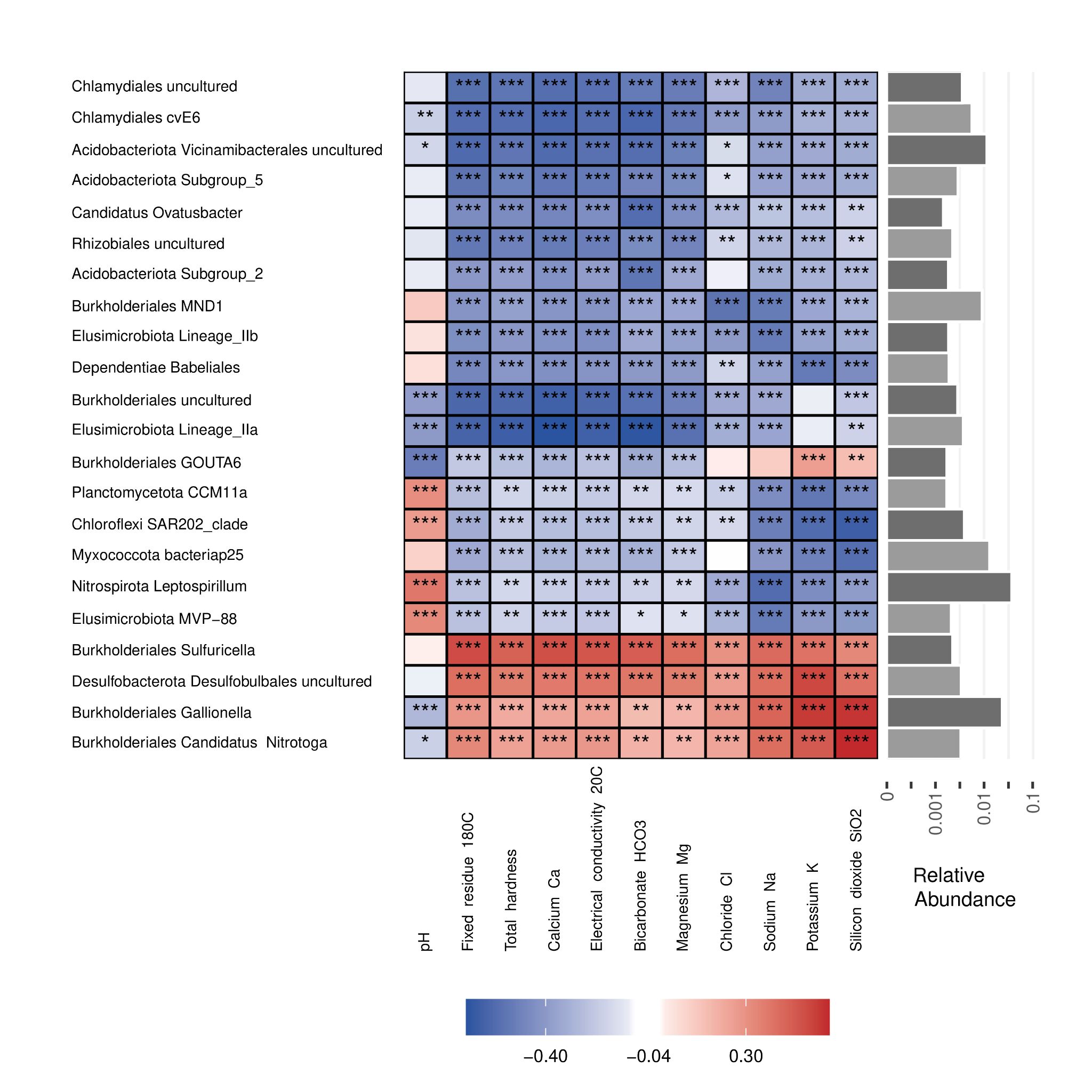
**

**Supplementary Figure 3.** Heatmap of the partial correlations for the presence of different genera against chemical parameters of spring water. The taxa displayed showed a Spearman correlation score bigger than 0.5 for at least one of the measured chemical entities and an average relative abundance greater than 0,1%.
